# Role of the new rural cooperative medical system in alleviating catastrophic medical payments for hypertension, stroke and coronary heart disease in poor rural areas of China

**DOI:** 10.1186/1471-2458-14-907

**Published:** 2014-09-02

**Authors:** Qi Wang, Huan Liu, Zu X Lu, Qing Luo, Jun A Liu

**Affiliations:** Department of Epidemiology and Biostatistics, School of Public Health, Tongji Medical College, Huazhong University of Science and Technology, No. 13, Hankong Road, Wuhan, 430030 Hubei Province China; Department of Social Medicine and Health Management, School of Public Health, Tongji Medical College, Huazhong University of Science and Technology, Wuhan, 430030 Hubei Province China

**Keywords:** Catastrophic impact, Out-of-pocket payment, Hypertension, Stroke, Coronary heart disease, New rural cooperative medical system

## Abstract

**Background:**

Hypertension, stroke and coronary heart disease (CHD) are common diseases that impose a heavy burden on patients and their families, particularly on those living in poor areas. This study examined catastrophic medical payments faced by patients with these diseases and the effectiveness of the new rural cooperative medical system (NRCMS) at alleviating the impact of the said diseases in fourth-class rural areas (i.e. those with annual income of less than RMB1500/$240.2 per capita) of China.

**Methods:**

Data on medical payments, including out-of-pocket and NRCMS-reimbursed expenses were collected through self-administered questionnaires. The pre- and post-reimbursement (via the NRCMS) prevalence of household poverty, catastrophic medical payment (CMP) incidence (H_*cat*_), mean CMP gap (G_*cat*_), mean positive CMP gap (MPG_*cat*_) and other determinants of CMP incidence were identified.

**Results:**

Out-of-pocket payments for treatment of hypertension, stroke and CHD averaged RMB580.1/$92.9, RMB3028.4/$484.8 and RMB1561.4/$250.0 per capita, respectively, in 2008. H_*cat*_, G_*cat*_ and MPG_*cat*_ due to the three diseases were 17.0%, 16.6% and 97.6%, respectively, and reimbursement through the NRCMS reduced them to 13.5%, 11.8% and 87.4%, respectively. The difference between pre- and post-reimbursement H_*cat*_ was not statistically significant. After adjusting the covariates for age [*OR* = 1.87, 95% confidence interval (*CI*) = 1.19-2.95], education (*OR* = 1.56, 95% *CI* = 1.07-2.27), marital status (*OR* = 1.67, 95% *CI* = 1.11-2.51), occupation (*OR* = 1.96, 95% *CI* = 1.34-2.85), annual income (*OR* = 4.95, 95% *CI* = 3.28-7.48), the multiple logistic regression analysis revealed that patients with stroke (*OR* = 3.94, 95% *CI* = 2.38-6.51) or CHD (*OR* = 2.25, 95% *CI* = 1.38-3.65) were more susceptible to CMP compared with patients with hypertension only.

**Conclusions:**

Out-of-pocket medical spending on hypertension, stroke and CHD imposes a heavy financial burden on the residents of fourth-class rural areas of China. The NRCMS has some impact on reducing catastrophic medical payments associated with these diseases, but improvement of the reimbursement rate is necessary to further improve its effectiveness.

## Background

Hypertension is a highly prevalent disease associated with increased risk of cardiovascular diseases and high mortality rates worldwide. For this reason, it is one of the major public health concerns not only in the West but also in the Asia Pacific region [1]. Occurrence of hypertension among Chinese adults is on the rise due to economic development and concomitant changes in lifestyle, particularly in their diet. Between 2000 and 2001, 27.2% of Chinese adults aged 35 to 74 years, or about 129,824,000 individuals, had hypertension [2]. In 2002, 18% of adults aged 18 years and above, or about 153 million of the Chinese population, reportedly had hypertension [3]. Hypertension is also a major risk factor for stroke, coronary heart disease (CHD), heart failure, myocardial infarction and other serious cardiovascular and chronic kidney diseases. In a prospective cohort study involving 169,871 Chinese men and women aged 40 years and above, Gu et al. found a strong, linear and independent relationship between blood pressure levels and the risk of cardiovascular diseases in Chinese adults [4].

Providing clinical care for hypertension and its complications imposes great economic burden on patients as well as the country. According to the Chinese Third National Health Services Survey conducted in 2003, direct medical spending on outpatient services averaged RMB72.4/$11.6, RMB215.4/$34.5, and RMB177.3/$28.4 for each visit associated with hypertension, stroke, and CHD, respectively, in rural areas. Furthermore, direct medical spending on inpatient services averaged RMB1329.6/$212.9, RMB2799.1/$448.1, and RMB2108.4/$337.6 for each visit associated with hypertension, stroke, and CHD, respectively, in rural areas [5]. Lloyd-Jones et al. estimated the cost of hypertension in the US at $73.4 billion in 2009 [6]. According to Zhai et al., the direct medical costs of Chinese patients aged 35 to 74 years with hypertension, CHD and stroke in 2003 were RMB20.2/$3.23, RMB15.7/$2.51, and RMB24.3/3.89 billion, respectively. Similarly, medical cost attributed to hypertension was estimated at RMB19.1 billion/$3.06 billion, which accounts for 47.7% of the total medical cost of treatment of stroke and CHD [7].

Health spending is considered to be catastrophic when a household must reduce its basic expenditure over a period of time to cope with health costs, but there is no consensus on the threshold [8]. Therefore, catastrophic medical payment (CMP) reflects a household’s payment capacity. If a family’s out-of-pocket (OOP) health expenditure exceeds its total disposable income for a certain period by 30% or more, then the family is considered to be facing CMP. According to a report by Xu et al., in which a threshold of 40% was used, the incidence of CMP varies across countries; it is low in developed countries (e.g., 0.57% in Switzerland and 0.55% in the US) and high in countries experiencing economic transitions (e.g., 7.50% in Azerbaijan and 10.45% in Vietnam) [9]. Himmelstein found that CMP incidence is below 1% in some countries, but as high as 13% in other countries [10]. Notably, even wealthy families may be subjected to CMP [10].

Out-of-pocket payments are the principal means of financing healthcare in China, which has led to high incidence of CMP [11]. According to Li et al., rural households have greater risk of incurring CMP than urban households [12]. Likewise, CMP is observed more frequently in households located in poor areas than in those located in wealthy regions [12]. Hence, in rural China, particularly in the poorest regions with annual income of no more than RMB1500/$240.2 per capita, medical expenditure is an important cause of transient poverty [13]. Members of poor families usually suffer from chronic diseases such as hypertension and CHD that constitute the main reason for sickness poverty. According to the Third National Health Services survey conducted in China in 2003, the prevalence of common chronic diseases in rural areas was 12.6% [5]. Similarly, according to a study conducted by Ma et al. in southwest China, approximately 38.8% of poor rural families were victimized by chronic illnesses, with significantly higher proportion of impoverished families affected compared to non-impoverished families [14]. Ying et al. found that the mean self-paid (i.e. OOP) medical cost of poor families is RMB1414/$ 226.4 per month, which accounts for 70% of their average monthly expenditure [15]. In the case of a major illness, poor families may give up treatment or cut down their budget for other living necessities (e.g., food, clothing, housing and children’s education) and/or borrow money to afford medical costs, thereby increasing the severity of their poverty [16].

The three key prerequisites for CMP are poor access to health services, expensive medical costs and lack of health insurance [9]. Ramses and Karine Lamiraud pointed out that expanding the coverage of the medical insurance system could reduce the incidence of CMP [17]. To prevent the phenomenon of ‘poverty due to illness’ in rural China, the New Rural Cooperative Medical System (NRCMS) was introduced to rural health sectors in 2003. NRCMS is financed by combined contributions from central and local government bodies and from individual households [18]. NRCMS operates at the county level. Counties determine the deductible, ceiling, reimbursement ratio, medical saving account (MSA). The ministry of health of central and local government agencies is the policy maker and mainstay of NRCMS. Central and local governments initially contributed RMB40/$6.4 for each enrollee annually since 2003, whereas participants only contributed RMB10/$1.6. This criterion doubled both for central and local governments and individual participants in 2008. In practice, NRCMS reimburses approximately 30% of inpatient expenditures [19, 20]. There are essentially four types of benefit and reimbursement models under NRCMS. The most common model (especially in the western and central regions) is implemented in 47% of Chinese counties. This model involves formula-based reimbursement of inpatient services and the use of MSA for reimbursements of outpatient services and preventive care. Household are expected to make contribution to MSAs, and household members can use the MSA for outpatient services. A second model is similar (applied in some 41% of Chinese counties). It uses the same inpatient reimbursement policy, but there is no MSA. Use of these two models has changed rapidly, reflecting the policy to phase out MSAs gradually over time. In 2007, for example, nearly two-thirds of counties used the first model and 7% used the second. Outpatient services and preventive care can be reimbursed from pooled funds, and is subject to a formula but usually involves no deductible. The third model (applied in about 8% of Chinese counties) reimburses both inpatient and outpatient services for catastrophic diseases, with separate deductibles and reimbursement caps. The fourth model used in 4% of Chinese counties reimburses both inpatient and outpatient services from pooled funds. All four models emphasize reimbursement for inpatient services, which are subject to deductibles and caps. Inpatient deductibles were RMB100 (range 0–200)/$16.0 (range 0–32.0), RMB250 (range 0–500)/$40.0 (range 0–80.0), and RMB800 (range 0–2000)/$128.1 (range 0–320.0) at township, county, and provincial hospitals, respectively. The average reimbursement ceiling was RMB30,000 (range 8,000-180,000)/$4803.0 (range 1280.8-28818) for inpatient care [19].

By the end of September 2008, more than 95% of counties in China were covered by the program [21]. The NRCMS has become one of three major medical insurances in the country, with rural residents covered by the NRCMS, urban employees under the Urban Employees Basic Medical Insurance and unemployed urban residents under the Urban Residents Basic Medical Insurance. The main goal of the NRCMS is to improve access of rural residents to health services and, above all, to help them reduce the risks that accompany catastrophic illnesses, thereby protecting them from impoverishment caused by CMP [22, 23].

The present study examines the incidence and severity of catastrophic medical payments due to hypertension, stroke and CHD, and identifies the role of the NRCMS in alleviating CMP-induced poverty in four poor rural counties in central and western China.

## Methods

Face-to-face interviews were conducted on patients with hypertension and/or stroke and CHD in the Hong Kong Kadoorie Project. Self-administrated questionnaires were used to collect data on medical payments, including out-of-pocket (OOP) and NRCMS-reimbursed expenses. The pre- and post-reimbursement (via the NRCMS) prevalence of household poverty, catastrophic medical payment (CMP) incidence (H_*cat*_), mean CMP gap (G_*cat*_), mean positive CMP gap (MPG_*cat*_) and other determinants of CMP incidence were calculated.

The research protocol was approved by the Ethics Committee of Tongji Medical College, Huazhong University of Science and Technology (IRB No: FWA000007304). Informed consents were obtained from all participants prior to collection of the data.

### Study area and subjects

The research was conducted based on data collected from an investigation carried out between October 15 and October 30 in 2009. The investigation took place in areas where hypertension patients were all involved in the Hong Kong Kadoorie Project of ‘Community Health Promotion in Poor Rural Areas of China’, which funds the systematic management of prevalent hypertension and its complications in rural districts of central and western China. Under the Kadoorie Project, local general practitioners are trained to diagnose hypertension and its complications, perform regular interviews, provide guidelines for drug use and offer health education programs to patients. This project differs from health insurance (e.g., NRCMS) and does not provide the patients with any economic help. A total of 10 towns—three in the Ledu county of Qinghai Province, three in the Hezheng county of Gansu Province, one in the Jiaocheng county and three in the Pinglu county of Shanxi Province—were involved in this investigation. All of them were classified as fourth-class rural areas where the average annual income was less than RMB1500/$240.2 per capita in 2003. In other words, these rural areas have the worst economic level among all Chinese rural districts [13]. At the time of the investigation, the four counties (i.e. Ledu, Hezheng, Jiaocheng and Pinglu) had total populations of 209,046, 166,586, 158,331 and 213,912, respectively, and an average annual income of RMB3130/$501.1, RMB1727/$276.5, RMB2920/$467.5 and RMB1440/$230.5 per capita, respectively. Cluster sampling strategy was used to randomly select two villages in each of the 10 towns.

All of the 1528 hypertension patients in the 20 selected villages were involved in the Kadoorie Project. According to the reported 12.6% prevalence of hypertension among adults aged 18 years or above in fourth-class rural regions, about 2500 prevalent hypertension cases existed in all of the 20 villages (with a total of 20,000 adults aged 18 years or above) [24]. However, some of the patients had left their hometown for residence or work in other cities. Only 1528 hypertension patients were left in the study area, and only 1262 of them from 1176 households participated in our investigation. The other 266 patients were not interviewed because of various reasons, such as leaving home for work and inconvenient communication. More than 95% of the rural residents in the selected areas were NRCMS participants.

In this study, hypertension was defined as systolic blood pressure of ≥ 140 mmHg, diastolic blood pressure of ≥ 90 mmHg and/or a self-reported current treatment for hypertension. All subjects were aged 21 to 90 years (with a mean of 61.2 years) and were diagnosed with hypertension by a physician in township health centres or reputed hospitals. The respondents consisted of 1000 patients with hypertension from 947 families, 112 patients with stroke from 110 families, and 150 patients with CHD from 149 families. Patients who were suffering from secondary hypertension were excluded from the count.

### Data collection

Self-administered questionnaires were used for collecting data on sociodemographic characteristics including gender, age, nationality (i.e. Han or others such as Hui, Uygur, Dongxiang, Tujia or Kazak), marital status (i.e. married, divorced, widowed or unmarried), educational background (i.e. elementary, middle or senior middle school), occupation (i.e. farming or non-farming), and family status (i.e. general or enjoying five guarantees (i.e. childless and infirm; old persons who are guaranteed food, clothing, medical care, housing and burial expenses by communes; low-income families)), household income and household medical expenditures for treatment of hypertension, stroke, CHD and other diseases (including costs of outpatient and inpatient services) that were disbursed through either OOP or the NRCMS (reimbursement) in the preceding year (i.e. 2008). The economic status of households was evaluated by comparing the annual per capita income with the poverty line of RMB1300/$208.1 per capita set by the Chinese State Council Poverty Alleviation Leading Group Office; a poor economic status indicates an annual per capita income below the threshold, and a non-poor economic status indicates an annual per capita income equal to or above the threshold.

Most of the participants were recruited to the village health care centres by local general practitioners. Face-to-face interviews were conducted by trained investigators. All completed questionnaires were returned on the spot. Some of the participants who experienced difficulties in walking received the interview and completed the questionnaire at their own homes with the help of our investigators.

### Data analysis

#### Prevalence of household poverty

The Chinese State Council Poverty Alleviation Leading Group Office set the poverty line at an annual income of RMB1300/$208.1 per capita. Families with income less than this amount are considered poverty-stricken families in the present study. The prevalence of household poverty (H_*pov*_) was calculated using the formula below, where N represents the sample size of families and P is designated a value of 0 or 1, which indicates whether a household is poverty-stricken or not.


A household’s poverty status can be evaluated by H_*pov*_. Impoverishment (expressed as H_*H-pov*_) as a result of health costs related to hypertension, stroke or CHD was estimated by subtracting the medical costs prior to NRCMS reimbursements from the household income. Thus, the difference between H_*pov*_ and H_*H-pov*_ indicates the extent to which the health costs of the studied diseases impoverish the households. H_*H-pov*_ was also calculated after the NRCMS reimbursements. The role of the NRCMS in alleviating impoverishment induced by the studied diseases can thus be evaluated quantitatively using the difference between pre- and post-reimbursement (via the NRCMS) H_*H-pov*_s.

#### Incidence and severity of catastrophic medical payments

The incidence and intensity of CMP were measured as described previously by Wagstaff and van Doorslaer MP [25].

An OOP medical payment is considered financially catastrophic when it is large relative to the household’s ability to pay (e.g., exceeding 15% of the family income) [8]. Thresholds of 10%, 20%, 30%, 40%, 50% and 60% of household capacity to pay (CTP) were used to define CMP. In this study, a threshold of 40% was adopted [8]. The World Bank and the WHO define CTP as the household’s disposable income calculated as the total income minus subsistence expenditure [25]. CTP can be evaluated based on household consumption, expenditure, income or wealth index. Household income was used to evaluate CTP in this study. In the following description, X represents CTP, T represents payments for healthcare and z_*cat*_ represents the threshold of CMP, which amounts to 40%. Oi is the catastrophic overshoot, which is equal to Ti/xi-z_*cat*_ if Ti/xi > z_*cat*_ and to 0 if otherwise; Ei is equal to 1 if Oi > 0 and to zero if otherwise.

CMP incidence describes the frequency of CMP in all the studied households. It is calculated from the formula below, where H_*cat*_ is the CMP incidence, and N is the sample size of families.


CMP incidence reflects the number of families with catastrophic medical spending (i.e. the number of families with health payments that go beyond the predetermined threshold). However, it cannot represent how much a given household pays beyond its CTP. Another parameter of mean CMP gap was thus used to measure catastrophic severity. The CMP gap describes how much of a household’s medical payment is in excess of the threshold of 40% of its CTP. The mean CMP gap can be calculated from the equation below, where G_*cat*_ is the mean CMP gap, and N is the sample size of all selected families.


G_*cat*_ is estimated to reveal the average level of CMP severity for all studied households, but it cannot indicate to what extent excessive payments affect the said households. This value can be illustrated by the parameter mean positive CMP gap (MPG_*cat*_), which refers to the average excess payment made by households in the sample, and is calculated as follows:


#### Statistical analysis

EpiData version 3.1 was used to manage all data. Chi-squared test was used to analyse the demographics of the subjects among the three groups (i.e. hypertension, stroke, and CHD groups). Multiple logistic regression analysis was performed with a multilevel model because both individual- and family-level variables are potential determinants of CMP incidence caused by hypertension, stroke and CHD. Statistical analysis was conducted using SAS version 8.12.

## Results

### Demographics and socio-economic status of the subjects

The demographics and socio-economic status of all the subjects are shown in Table 1. The mean (SD) age of the subjects was 61.2 (12.0) years. Among the subjects, 37.4% were male (n = 472) and 62.6% were female (n = 790). Most of them (90.1%) were of Han ancestry. The average annual income of the subjects was RMB2263.7/$362.4 per capita. About 42.5% of the subjects came from families that were below the poverty line set by the Chinese State Council Poverty Alleviation Leading Group Office. Village clinics (47.8%) and township health centres (47.7%), which were about 30 minutes away from the residences of 85.3% of the subjects, were the main and nearest providers of medical care services. Over 98.9% of the subjects were covered by a medical insurance system, and most of the subjects (98.0%) took part in the NRCMS.

**Table 1:** **Demographics and social economics of all subjects enrolled in the study**

Variables		Hypertension	Stroke	***CHD***	***Total***	***Statistics***
**Family member aged 60 years or above**	Yes	638	91	117	846	*χ2* = 22.97, *P* < 0.001
	No	361	21	33	415	
**Family status**	General	924	90	139	1153	*χ2* = 18.88, *P* < 0.001
	Others^†^	76	22	11	109	
**Occupation**	Farming	562	60	102	724	*χ2* = 8.15, *P* = 0.017
	Non-farming	438	52	48	538	
**Economic status** ^**△**^	Non-poor	591	62	73	726	*χ2* = 6.05, *P* = 0.049
	Poor	409	50	77	536	
**Nearest medical centre**	Village clinic	462	48	97	607	*P* = 0.001 (Fisher)
	Township health centre	485	60	47	592	
	County hospital	5	1	1	7	
	Personal clinic	48	3	5	56	
**Time spent travelling to the nearest medical centre**	Less than 30 min.	858	89	122	1069	*P* = 0.241 (Fisher)
	30 min. to 60 min.	110	18	23	151	
	over 60 min.	32	5	5	42	
**Age**	Less than 60 years	485	23	50	558	*χ2* = 40.11, *P* < 0.001
	60 years and above	515	89	100	704	
**Gender**	Male	368	55	49	472	*χ2* = 8.15, *P* = 0.017
	Female	632	57	101	790	
**Race**	Han	909	105	123	1137	*χ2* = 13.42, *P* = 0.001
	Others^‖^	91	7	27	125	
**Educational background**	Illiterate	350	51	64	465	*χ2* = 7.28, *P* = 0.026
	Others^*^	650	61	86	797	
**Marital status**	Married	820	85	112	1017	*χ2* = 6.21, *P* = 0.045
	Others^※^	180	27	38	245	
**Medical insurance**	Yes	990	112	147	1249	*P* = 0.230 (Fisher)
	No	10	0	3	13	

^†^Households enjoying five guarantees (i.e. childless and infirm; old persons who are guaranteed food, clothing, medical care, housing and burial expenses by communes; low-income families).

^△^The economic status of households was evaluated by comparing the annual per capita income with the poverty line of RMB1300/$208.1 per capita set by the Chinese State Council Poverty Alleviation Leading Group Office; a poor economic status indicates an annual per capita income below the threshold, and a non-poor economic status indicates an annual per capita income equal to or above the threshold.

^‖^Nationality of Hui, Uygur, Dongxiang, Tujia, or Kazak etc. except Han.

^*^Elementary, middle or senior middle school.

^※^Divorced, widowed or unmarried.

### Household expenditure on all health services

Household expenditures on health services by the subjects are shown in Table 2. Poor families spent 32.0% of their household income for health services, whereas non-poor families spent only 23.5%. This difference is statistically significant (*χ2* = 13.82, *P* = 0.0002). In 2008, the mean health cost of hypertension, stroke and CHD among poor families was RMB873.1/$139.8 per capita, which was not significantly lower than the RMB946.8/$151.6 spent by non-poor families (*χ2* = 0.72, *P* = 0.3971). Subjects from poverty-stricken families were found to have mean expenditures of RMB583.7/$93.5, RMB2808.4/$449.6 and RMB1153.4/$184.7, which corresponded to 24.7%, 65.4% and 31.5% of total household health expenditure, for hypertension, stroke and CHD, respectively, in 2008. The corresponding values for other subjects were RMB577.6/$92.5 (15.7%), RMB3205.7/$513.2 (49.9%) and RMB1991.8/$318.9 (42.7%) for hypertension, stroke and CHD, respectively. However, the differences between poverty-stricken families and others were not statistically significant.

**Table 2:** **Household health expenditures among the families of the subjects**

Diseases	Family type ^△^	N ^*^ / Fa-N ^†^	HE _***HypD***_^‡^ (RMB/$)	HE _***T***_^§^ (RMB/$)	HE _***HypD***_/HE _***T***_(%)	IHE _***HypD***_^||^ (RMB/$)
**Hypertension**	Non-poor	591/558	341356/54651	2175912/348364	15.7	577.6/92.5
	Poor	409/389	238729/38221	966567/154747	24.7	583.7/93.5
	Total	1000/947	580085/92872	3142479/503111	18.5	580.1/92.9
**Stroke**	Non-poor	62/60	198754/31821	398600/63816	49.9	3205.7/513.2
	Poor	50/50	140422/22482	214890/34404	65.4	2808.4/449.6
	Total	112/110	339176/54302	613490/98220	55.3	3028.4/484.8
**CHD**	Non-poor	73/73	145399/23278	340310/54484	42.7	1991.8/318.9
	Poor	77/76	88812/14219	281700/45100	31.5	1153.4/184.7
	Total	150/149	234211/37497	622010/99584	37.6	1561.4/250.0
**Total**	Non-poor	724/684	685509/109750	2914822/466663	23.5	946.8/151.6
	Poor	536/502	467963/74921	1463157/238494	32.0	873.1/139.8
	Total	1262/1176	1153472/184671	4377979/713610	23.6	914.0/146.3

^**△**^A non-poor family type has an annual income of RMB1300/$208.1 per capita or above; a poor family type has an annual income of less than RMB1300/ $208.1 per capita.

^*^N indicates the number of subjects studied.

^†^Fa-N indicates the number of families of the subjects.

^‡^HE_*HypD*_ indicates the household expenditure related to hypertension, stroke and CHD among the studied families in the past year (2008).

^§^HE_*T*_ indicates the total household health expenditures of the studied families in the past year (2008).

^||^IHE_*HypD*_ indicates individual health expenditures related to hypertension, stroke and CHD in the past year (2008).

### Household poverty caused by out-of-pocket payments related to hypertension, stroke and CHD

The results for families impoverished by costs related to hypertension, stroke and CHD and the effects of NRCMS reimbursement are shown in Table 3. The NRCMS managed to decrease the poverty impact of OOP payments due to hypertension, stroke and CHD to 1.2% (from 6.9% pre-reimbursement to 5.7% post-reimbursement). The H_*pov*_ values for families with hypertension, stroke or CHD patients were not significantly different (*χ2 =* 5.636*, P =* 0.060). The NRCMS managed to lower the poverty impact of OOP payments due to hypertension to 0.4% (from 4.7% to 4.3%), that due to stroke to 4.5% (from 6.4% to 1.9%), and that due to CHD to 3.3% (from 13.4% to 10.1%). Hypertension, stroke and CHD are the major chronic diseases that impoverish rural families in the study areas. The NRCMS alleviated the financial burden of these diseases on the households to some extent, but the effects were statistically insignificant.

**Table 3:** **Prevalence (%) of fundamental poverty and the pre- and post-reimbursement (via the NRCMS) impoverishment caused by medical payments for hypertension, stroke and CHD among the families under review**

Variables	Hypertension (n = 947)	Stroke (n = 110)	CHD (n = 149)	Total (n = 1176)	Statistics ^#^
**H**_***pov***_	40.9	44.5	51.0	42.5	*χ2 =* 5.636*, P =* 0.060
**Pre-reimbursement H**_***H-pov***_	45.6	60.9	64.4	49.4	*χ2 = 24.712, P < 0.001*
**Post-reimbursement H**_***H-pov***_	45.2	56.4	61.1	48.2	*χ2 = 16.251, P < 0.001*
**Declines**	0.4	4.5	3.3	1.2	
**Statistics** ^**^**^	*χ2 =* 0.019*, P =* 0.890	*χ2 =* 0.334*, P =* 0.563	*χ2 =* 0.469*, P =* 0.494	*χ2 =* 0.359*, P =* 0.549	

^#^The Pearson chi-squared test was used to compare the incidence of household poverty among families with members suffering from hypertension, stroke and CHD.

^^^The Pearson chi-squared test was used to compare the incidence of household poverty before and after NRCMS reimbursement.

### Incidence and severity of catastrophic medical payments

The results for incidence and severity of catastrophic medical payments are shown in Table 4. The H_*cat*_, G_*cat*_ and MPG_*cat*_ values of all the families were 44.4%, 66.4% and 149.6%, respectively. The H_*cat*_ values of the poor and non-poor families were 64.8% and 29.3%, respectively. Poor families were more likely to be subjected to CMP compared with non-poor families (*χ2* = 146.82, *P* < 0.001). The corresponding G_*cat*_ and MPG_*cat*_ of the poor families were 131.3% and 202.7%, respectively, whereas those of the non-poor families were 18.4% and 62.9%, respectively.

**Table 4:** **Results of the incidence and severity of catastrophic medical payments among all the studied families**

Variables	Poor families ^†^	Non-poor families ^‡^	Total
**N. of family** ^*^	324	198	522
**H**_***cat***_ **(%)** ^**#**^	64.8	29.3	44.4
**G**_***cat***_ **(%)**	131.3	18.4	66.4
**MPG**_***cat***_ **(%)**	202.7	62.9	149.6

^†^Poor families indicate those with an annual income of less than RMB1300/$208.1 per capita.

^‡^Non-poor families indicate those with an annual income of RMB1300/$208.1 per capita or above.

^*^N. of family indicates the number of families with CMP.

^**#**^Statistics: *χ2 =* 146.82*, P <* 0.001.

Our findings on the incidence and severity of catastrophic medical payments associated with hypertension, stroke and CHD are shown in Table 5. H_*cat*_ was 17.0% due to the costs related to the three diseases. The NRCMS managed to lower CMP incidence to 13.5%. The representative H_*cat*_ values for hypertension, stroke and CHD were 12.4%, 40.9% and 26.8%, respectively, and the NRCMS lowered these values to 9.7%, 30.9% and 20.8%, respectively. Thus, the NRCMS had an important role in easing the economic pressure and decreasing the incidence and severity of catastrophic medical payments caused by the three diseases. However, the decrease in CMP incidence produced by the NRCMS was fairly small. The G_*cat*_ and MPG_*cat*_ values associated with stroke were larger than those associated with hypertension and CHD, suggesting that stroke imposes a larger financial burden on families than the other two diseases.

**Table 5:** **Results of the pre- and post-reimbursement (via the NRCMS) catastrophic medical payments related to hypertension, stroke and CHD**

Variables	Hypertension	Stroke	CHD	Total
**N. of families** ^*^	114 vs 92	45 vs 34	40 vs 31	199 vs 157
**H**_***cat***_ **(%)**	12.4 vs 9.7	40.9 vs 30.9	26.8 vs 20.8	17.0 vs 13.5
**G**_***cat***_ **(%)**	7.4 vs 5.3	77.3 vs 58.8	25.4 vs 15.1	16.6 vs 11.8
**MPG**_***cat***_ **(%)**	61.6 vs 54.6	190.4 vs 188.9	94.8 vs 72.6	97.6 vs 87.4
**Statistics** ^**#**^	*χ2 =* 2.636*, P =* 0.104	*χ2 =* 2.390*, P =* 0.122	*χ2 =* 1.498*, P =* 0.221	

^*^N. of families indicates the number of families with CMP for hypertensive diseases.

^**#**^The Pearson chi-squared test was used to compare H_*cat*_ before and after the NRCMS reimbursement for the medical costs related to the studied diseases.

### Influential factors associated with CMP incidence among individuals

Multiple logistic regression analysis with forward selection was used to determine the factors related to CMP incidence resulting from hypertension, stroke or CHD among the studied individuals. The outcome variable of this process was the status of CMP incidence (yes or no), and the independent variables included gender, age, race, educational background, marital status, occupation, income, prevalent disease (hypertension, stroke or CHD), time spent travelling to the nearest medical centre, medical insurance type and the nearest medical centre. The results are shown in Table 6. The following subjects were greatly susceptible to CMP, as seen in their corresponding representative *ORs* (95% *CIs*): those aged 60 years or above (relative to those aged 60 years and below), 1.87 (1.9-2.95); those with no educational background (relative to the non-illiterate), 1.56 (1.07-2.27); those whose marital status was divorced, widowed or unmarried (relative to married participants), 1.67 (1.11-2.51); those with a non-farming occupation (relative to those with a farming occupation), 1.96 (1.34-2.85); those with an annual income of less than RMB1300/$208.1 per capita (relative to those with an annual income of RMB1300/$208.1 or above per capita), 4.95 (3.28-7.48); those suffering from stroke (relative to those suffering from hypertension only), 3.94 (2.38-6.51); those suffering from CHD (relative to those suffering from hypertension only), 2.25 (1.38-3.65).

**Table 6:** **Results of the multiple logistic regression analysis determining the factors associated with the post- reimbursement (via the NRCMS) CMP incidence caused by medical payments for hypertension, stroke or CHD** ^*^

Variables	Values	***OR (95% CI)***	***P***
Age	60 years or below	1	
	60 years or above	1.87 (1.19, 2.95)	0.0067
Educational background	Literate	1	
	Illiterate	1.56 (1.07, 2.27)	0.0214
Marital status	Married	1	
	Divorced, widowed or unmarried	1.67 (1.11, 2.51)	0.0145
Occupation	Farming	1	
	Non-farming	1.96 (1.34, 2.85)	0.0004
Annual income	RMB1300/$208.1 per capita or above	1	
	Less than RMB1300/$208.1 per capita	4.95 (3.28, 7.48)	<0.0001
Disease	Hypertension	1	
	Stroke	3.94 (2.38, 6.51)	<0.0001
	CHD	2.25 (1.38, 3.65)	0.0011

*-2 Log L (intercept and covariates) = 783.026; R-square = 0.1226; max-rescaled R-square = 0.2320; Wald chi-square = 129.56, *P* < 0.001; Deviance value = 459.8351 (df = 527), *P* = 0.9839; Pearson value = 633.3337 (df = 527), *P* = 0.0010.

## Discussion

Rural households in central and western China, particularly those below the poverty line, are always susceptible to disease-induced poverty [13]. Even a fairly small medical cost can be catastrophic for them. According to our findings, poor families (i.e. those with an average income of less than RMB1300/$208.1 per capita in 2008) had smaller medical expenditures related to hypertension, stroke and CHD in 2008 than non-poor families (i.e. those with an average income of RMB1300/$208.1 or above per capita in 2008). In addition, the proportion of health costs to total household health expenditure for hypertension, stroke and CHD was significantly higher in the former than in the latter. Of the three diseases we focused on, we found stroke to incur the largest medical expenditures, which is consistent with a previous report by Zhai et al. [7].

Over 40% (44.4%) of the families were found to have suffered from catastrophic medical payments, with a G_*cat*_ of 66.4% and an MPG_*cat*_ of 149.6%. The poor families included in the study had significantly higher CMP incidence, G_*cat*_ and MPG_*cat*_ than non-poor families. According to our investigation, some of the families enduring CMP needed to sell personal items to pay for their health expenditures (data not shown). Hypertension, stroke and CHD were also discovered to be important causes of CMP. CMP incidence resulting from these diseases was 17.0%, which was lowered to 13.5% via NRCMS reimbursement. Moreover, G_*cat*_ and MPG_*cat*_ were 16.6% and 97.6%, respectively, and dropped to 11.8% and 87.4%, respectively, after NRCMS reimbursement. In addition, the NRCMS managed to curtail the poverty impact of OOP payments due to hypertension, stroke and CHD to 1.2% (from 6.9% pre-reimbursement to 5.7% post-reimbursement). Thus, our findings showed that NRCMS reimbursements led to small decreases in the incidence and severity of catastrophic medical payments for hypertension, stroke and CHD, which suggests that the NRCMS plays a limited role in alleviating CMP-induced poverty among poor rural families [26].

The NRCMS policy focuses mainly on decreasing OOP payments for inpatient services to provide rural residents, particularly the disadvantaged ones, with easy access to health services and to reduce household risk of catastrophic medical payments resulting from major diseases. Thus, inpatient service utilisations increased under the NRCMS, whereas outpatient service utilisations did not [27, 28]. According to our investigation (data not shown), the OOP costs of outpatient service constituted a larger part (mean of 85.8%) of the total medical costs of hypertension, stroke, and CHD, compared with that of inpatient service (mean of 14.2%). Moreover, the NRCMS reimbursement rate to outpatient service averaged 7.3% (median 0.0%, and range 0–100.0%) and 54.6% (median 60.0%, and range 0–100.0%) for the 1005 patients receiving outpatient services and the 207 patients receiving inpatient services, respectively, for hypertension, stroke, and CHD in the past one year (i.e. 2008). Additionally, approximately 73.9% of the participants reported medical costs almost equal to or beyond their acceptable level in our research (data not shown). Thus, our findings suggest that the NRCMS did not sufficiently relieve poverty caused mainly by the purchase of anti-hypertension drugs, which is highly prevalent in outpatient services. Moreover, the goal of the NRCMS to provide insurance for rural residents against catastrophic illnesses has not been achieved [29]. The present proportion of OOP medical payments is fairly high for rural households in China [30]. Since most health payments in China comprise high percentages of OOP payments, China is more susceptible to CMP than other countries. CMP is highly unlikely to occur in families with OOP health payments lower than 15% [31].

Poverty among rural families also merits close attention. CMP is not caused simply by expensive medical procedures or interventions. Even a relatively small payment may mean a financial catastrophe to poor households, forcing them to cut down their budget for basic expenses such as food, shelter or children’s education [32]. Local poverty may lead to decrease in the number of people who seek medical care when they are sick and may subsequently affect accessibility of health services [16]. Many studies have shown that CMP is very likely to occur in households with poor economic conditions [9, 33–35]. The results of our logistic regression analysis show that household economic conditions are associated with CMP incidence, i.e. poor families are more likely to be subjected to CMP compared with non-poor families. Prevention of CMP can therefore lead to increase in the income of rural households, particularly of poor ones. Poor households can be protected from CMP by reducing the reliance of the health system on OOP costs, providing them with additional financial security, such as medical finance assistance, and increasing the reimbursement rate of the NRCMS.

Stroke and CHD are common complications of hypertension. Unlike patients suffering from hypertension only, patients with stroke or CHD are very susceptible to CMP. Therefore, prevention of hypertension complications is also necessary to decrease CMP incidence. Lowering the price of anti-hypertension drugs may make the drugs accessible to patients and in turn decrease their risk for hypertension-related complications. Medical insurance was not found to be associated with CMP, indicating that NRCMS reimbursements do not efficiently decrease the impacts of OOP costs of hypertension, stroke and CHD on patients. Furthermore, the low reimbursement rate of outpatient services may also be a point of concern.

Given the possibility of catastrophic effects of medical payments for hypertension, stroke and CHD on the financial conditions of patients, a better mechanism for implementation of NRCMS is needed to improve the reimbursement procedures for inpatient services and drug purchase, and to lower the burden of OOP costs on patients. Prevention and control of hypertension and its complications is a major public health issue. To some extent, the present NRCMS and Medical Assistance Policy have benefited hypertension patients and reduced the risk of consequential poverty among rural families in China. However, the benefits for poor households with patients suffering from chronic diseases (e.g., hypertension and its complications) seem very small. To achieve its goal of decreasing impoverishment caused by treatment costs for hypertension and its complications, the NRCMS should consider providing additional or even full payment. In some areas across China, a mode of secondary reimbursement of medical costs has been extended to poor patients suffering from hypertension or its complications. However, this is just a temporary solution and more needs to be done to achieve the goal of drastically reducing CMP incidence.

A major limitation of our study is the conservative extensionality of the conclusions we reached. As mentioned above, our study was conducted on subjects who are part of the project ‘Community Health Promotion in Poor Rural Areas of China’. Subjects suffering from hypertension or its complications are systematically managed in the project, so they do not constitute a representative sample. In this light, our findings should be interpreted cautiously. The skewed gender distribution (37.40% males versus 62.60% females) of the subjects in our study is also notable because most male adults leave their poor rural hometowns for high-paying jobs in cities, whereas most women stay at home looking after their children and elders. This phenomenon is common in most poor rural areas of central and western China.

## Conclusions

Out-of-pocket payments for treatment of hypertension, stroke and CHD impose a heavy burden on households in fourth-class rural areas of China. The NRCMS policy is somewhat effective at preventing disease-induced poverty among poor rural households in China, but needs to be revised by improving the reimbursement rate in order to produce better outcomes.
